# Is two better than one? Muscle vibration plus robotic rehabilitation to improve upper limb spasticity and function: A pilot randomized controlled trial

**DOI:** 10.1371/journal.pone.0185936

**Published:** 2017-10-03

**Authors:** Rocco Salvatore Calabrò, Antonino Naro, Margherita Russo, Demetrio Milardi, Antonino Leo, Serena Filoni, Antonia Trinchera, Placido Bramanti

**Affiliations:** 1 IRCCS Centro Neurolesi “Bonino-Pulejo” Messina; Messina, Italy; 2 Department of Biomedical, Dental Sciences, and Morphological and Functional Images, University of Messina; Messina, Italy; 3 Fondazione Centri di Riabilitazione Padre Pio Onlus; San Giovanni Rotondo, Italy; Universite de Nantes, FRANCE

## Abstract

Even though robotic rehabilitation is very useful to improve motor function, there is no conclusive evidence on its role in reducing post-stroke spasticity. Focal muscle vibration (MV) is instead very useful to reduce segmental spasticity, with a consequent positive effect on motor function. Therefore, it could be possible to strengthen the effects of robotic rehabilitation by coupling MV. To this end, we designed a pilot randomized controlled trial (Clinical Trial NCT03110718) that included twenty patients suffering from unilateral post-stroke upper limb spasticity. Patients underwent 40 daily sessions of Armeo-Power training (1 hour/session, 5 sessions/week, for 8 weeks) with or without spastic antagonist MV. They were randomized into two groups of 10 individuals, which received (group-A) or not (group-B) MV. The intensity of MV, represented by the peak acceleration (a-peak), was calculated by the formula (2π*f*)2A, where *f* is the frequency of MV and A is the amplitude. Modified Ashworth Scale (MAS), short intracortical inhibition (SICI), and H_max_/M_max_ ratio (HMR) were the primary outcomes measured before and after (immediately and 4 weeks later) the end of the treatment. In all patients of group-A, we observed a greater reduction of MAS (p = 0.007, d = 0.6) and HMR (p<0.001, d = 0.7), and a more evident increase of SICI (p<0.001, d = 0.7) up to 4 weeks after the end of the treatment, as compared to group-B. Likewise, group-A showed a greater function outcome of upper limb (Functional Independence Measure p = 0.1, d = 0.7; Fugl-Meyer Assessment of the Upper Extremity p = 0.007, d = 0.4) up to 4 weeks after the end of the treatment. A significant correlation was found between the degree of MAS reduction and SICI increase in the agonist spastic muscles (p = 0.004). Our data show that this combined rehabilitative approach could be a promising option in improving upper limb spasticity and motor function. We could hypothesize that the greater rehabilitative outcome improvement may depend on a reshape of corticospinal plasticity induced by a sort of associative plasticity between Armeo-Power and MV.

## Introduction

Spasticity is defined as a velocity-dependent increase in muscle tone due to the hyper-excitability of muscle stretch reflex [1]. Spasticity of the upper limb is a common condition following stroke and traumatic brain injury and needs to be assessed carefully because of the significant adverse effects on patient’s motor functions, autonomy, and quality of life [2].

Different pharmacological and non-pharmacological approaches are currently available for upper limb spasticity management, as physiotherapy (including magnetic stimulation, electromagnetic therapy, sensory-motor techniques, and functional electrical stimulation treatment) and robot-assisted therapy [3–4]. In this regard, several studies suggest robotic devices, including the Armeo® (a robotic exoskeleton for the rehabilitation of upper limbs), may help reducing spasticity by modifying spasticity-related synaptic processes at either the brain or spinal level [5–13], resulting in spasticity reduction in antagonist muscles through, e.g., a strengthening of spinal reciprocal inhibition mechanisms [11].

Growing research is proposing segmental muscle vibration (MV) as being a powerful tool for the treatment of focal spasticity in post-stroke patients [14–15]. Mechanical devices deliver low-amplitude/high-frequency vibratory stimuli to specific muscles [16–17], thus offering strong proprioceptive inputs by activating the neural pathway from muscle spindle annulospiral endings to Ia-fiber, dorsal column–medial lemniscal pathway, the ventral posterolateral nucleus of the thalamus (and other nuclei of the basal ganglia), up to the primary somatosensory area (postcentral gyrus and posterior paracentral lobule of the parietal lobe), and the primary motor cortex [18–19]. At the cortical network level, proprioceptive inputs can alter the excitability of the corticospinal pathway by modulating intracortical inhibitory and facilitatory networks within primary sensory and motor cortex, and affecting the strength of sensory inputs to motor circuits [20–22]. In particular, periods of focal MV delivered alone can modify sensorimotor organization within the primary motor cortex (i.e., can increase or decrease motor evoked potential—MEP—and short intracortical inhibition (SICI) magnitude in the vibrated muscles, while opposite changes occur in the neighboring muscles), thus reducing segmental hyper-excitability and spasticity [20–22].

While focal MV is commonly used to reduce upper limb post-stroke spasticity, there is no conclusive evidence on the role of robotic rehabilitation in such a condition [14–17,23–27]. A strengthening of the effects of neurorobotics and MV on spasticity could be achieved by combining MV and neurorobotics. The rationale for combining Armeo-Power and MV to reduce spasticity could lie in the summation and amplification of their single modulatory effects on corticospinal excitability [28]. Specifically, it is hypothesizable that MV may strengthen the learning-dependent plasticity processes within sensory-motor areas that are in turn triggered by the intensive, repetitive, and task-oriented movement training offered by Armeo-Power [29–30]. Such an amplification may depend on a sort of associative plasticity (i.e., the one generated by timely coupling two different synaptic inputs) between MV and Armeo-Power [31–33].

To the best of our knowledge, this is the first attempt to investigate such approach. Indeed, a previous study combining MV with conventional physiotherapy used Armeo only as evaluating tool [14].

The aim of our study was to assess whether a combined protocol employing MV and Armeo-Power training, as compared to Armeo-Power alone, may improve upper limb spasticity and motor function in patients suffering from a hemispheric stroke in the chronic phase. To this end, we compared the clinical and electrophysiological after-effects of Armeo-Power with or without MV on upper limb spasticity. We also assessed the effects on upper limb motor function and muscle activation, disability burden, and mood, given that spasticity may have significant negative consequences on these outcomes. Further, it is important to evaluate mood, as it may negatively affect functional recovery [34–36], increase mortality [37], and weaken the compliance of the patient to the rehabilitative training [38–39].

## Materials and methods

### Design

We consecutively included all the eligible patients affected by stroke who were attending the Neurorobotic Rehabilitation Laboratory of the IRCCS Centro Neurolesi “Bonino-Pulejo” (Messina, Italy), from January 2015 to June 2015 (Clinical Trial: NCT03110718). See S1 and S2 Files for trial study protocol.

The study was designed as a pilot randomized controlled trial using a double-blind, parallel-group study design. The enrolled patients were randomly assigned to receive Armeo-Power paired with real MV (group-A) or Armeo-Power with sham MV (group-B) using an automated computer randomization program. The patients, the clinical assessors (who were different from the physiotherapist who managed Armeo-Power and MV), and the statisticians (who differed from the clinical assessors) were blinded to group allocation.

**Fig 1:**
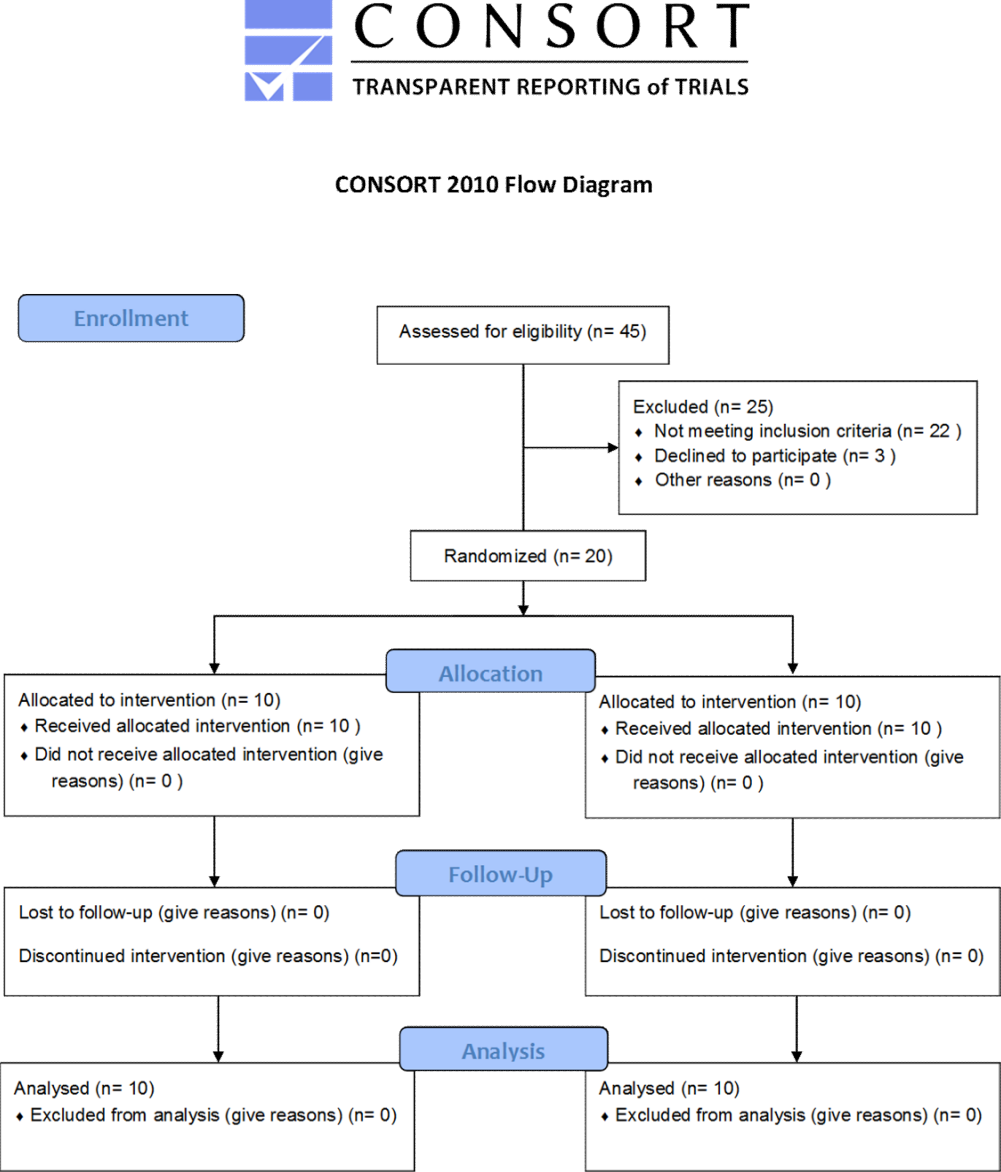
CONSORT flow diagram.

Twenty patients were included in this pilot study according to inclusion criteria as follows: a first ever supra-tentorial unilateral (left hemisphere) ischemic stroke experienced more than 3 months before the enrollment; a deficit of shoulder abductor, arm flexor, and elbow extensor muscles ranging from 2 to 4 on the Medical Research Council scale [40–41]; a spasticity of biceps brachii, pectoralis major, and latissimus dorsi (namely, spastic agonist muscles) ranging from 1+ to 3 on the Modified Ashworth Scale (MAS) [42–43]; ages between 50 and 80 years old; and Caucasian ethnicity. We excluded the patients who had history of concomitant neurodegenerative diseases or brain surgery; severe cognitive or language impairment; systemic, bone, or joint disorders; changes in central or peripheral sensitivity; concomitant use of drugs for spasticity; or contraindications to transcranial magnetic stimulation (TMS). CONSORT flowchart is reported in Fig 1; see S1 Table for CONSORT checklist.

The Local Ethics Committee approved the study and the procedures for gaining consent (study number registration 43/2013), and all the participants gave their written informed consent to the study. In comparison to the original trial study protocol (see supporting information), we lengthened the duration and number of rehabilitative sessions, and the epochs of assessment of clinical and electrophysiological outcomes, which were adapted to the aims and scope of the present pilot randomized clinical trial.

### Interventions

The Armeo-Power^a^ is a robotic, ergonomic arm exoskeleton for rehabilitation that cradles the entire arm, from shoulder to the hand (thus allowing intensive, repetitive, and task-oriented training of shoulder, elbow, wrist, and grasping movements) and counterbalances the weight of the patients’ arm thanks to a gravity-support system (offered by the arm exoskeleton). Armeo-Power allows the treatment of motor function impairment by enhancing any residual function and neuromuscular control, assisting active movement across a large 3D workspace, and providing augmented feedback [5–8,44].

All the patients underwent a daily Armeo-Power training session lasting about one hour, scheduled five times a week for eight consecutive weeks (for a total of 40 sessions). During the first session, the device was adjusted to the patient’s arm size and the angle of suspension. The working space and the exercises were selected once the upper limb had been fitted with the system. Subjects performed repetitively a customized group of exercises under the supervision of a skilled physiotherapist. Such exercises required all the available arm and elbow movements to improve shoulder abduction, arm flexion, and elbow extension (e.g., to parry penalties, collect drops of water with a cup, take the apples and place them in the shopping cart, clean surfaces, clean the stove, and break an egg into the pan). Device guidance force and arm weight support were individually adapted during the Armeo-Power training. The device automatically recorded information about the exercise (including the scheduled difficulty level, the score obtained, the time required to perform the exercise, the force exerted by the patient, and the passive and active range of movement).

**Fig 2:**
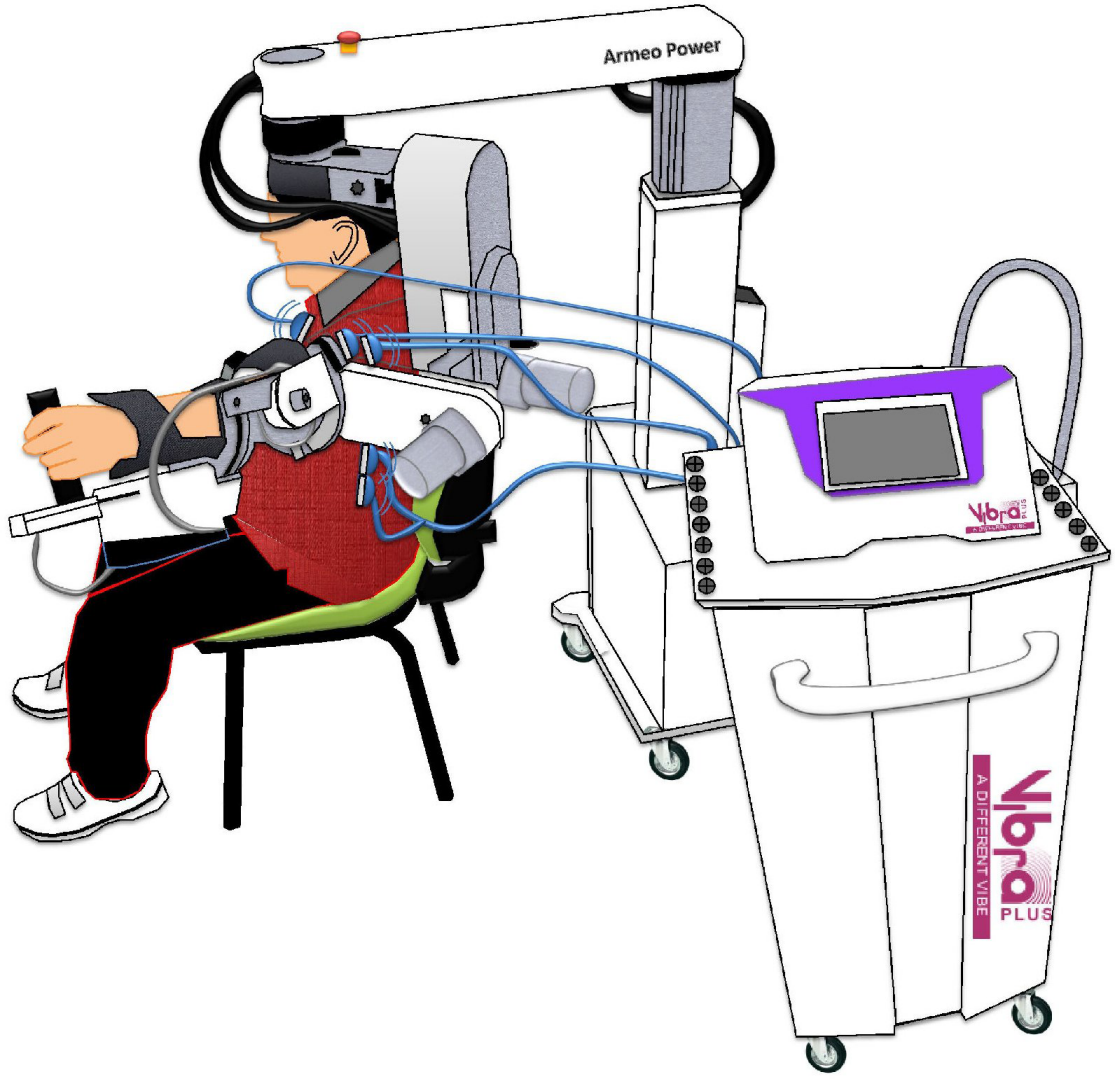
Combined rehabilitative approach.

All the subjects assigned to group-A received a focal belly MV on the spastic antagonist muscles (i.e., triceps brachialis, supraspinatus, and deltoid) during shoulder abduction and elbow extension (Fig 2).

MV was delivered by a pneumatic vibrator powered by compressed air^b^, and wired to probes with appropriate muscle diameter (up to 2cm^2^). The intensity of MV, represented by the peak acceleration (a-peak), was calculated by the formula (2π*f*)2A, where *f* is the frequency of MV (set at 80 Hz) and A is the amplitude of vibration (i.e., of the peak-to-peak sinusoidal displacement of the underneath structures), which was individually adapted (0.3±0.1 mm) to be just below the threshold for perceiving an illusory movement. We chose such a set up to avoid any signs of muscle contraction potentially reflecting either possible voluntary movement or occurrence of the tonic vibration reflex. Sham MV intensity was set at the same frequency but at 0.1 mm below the individually adjusted amplitude for real MV. MV parameter were kept constant throughout the treatment.

### Primary outcome measures

Patients were assessed at baseline (T0), directly after the neurorehabilitative training (T1), and after one month of rest from Armeo-Power training (T2) (during which the participants underwent a standard physical therapy treatment).

The primary outcomes consisted of the MAS from spastic agonist muscles (causing arm adduction, inward rotation, flexion, and forearm flexion), the SICI, and the Hmax/Mmax ratio (HMR).

The MAS measures spasticity, evaluating the resistance of a relaxed limb to a rapid passive stretch in six stages [45–46]. A score of zero indicates a normal or slightly increased muscle tone, whilst five indicates a state in which the passive movement is impossible. We tested the abduction–adduction, the flexion–extension, the intrarotation–extrarotation of the shoulder, and the flexion and extension of the elbow.

SICI is a measure of cortical excitability, given that it probes intracortical GABAergic interneurons within the primary motor area. It is tested by quantifying the inhibitory effect of a TMS pulse (conditioning) preceding of few milliseconds that eliciting MEP (test stimulus). SICI is typically reduced (i.e., values going toward 100%) in spasticity, maybe because of a deteriorated interhemispheric inhibition following brain damage. There is a significant correlation between spasticity and SICI given that baclofen has been shown to reduce spasticity by increasing GABAergic inhibition [47–48]. Consequently, one would expect a strengthening (i.e., a decrease) of intracortical inhibition when spasticity improves (i.e., the MAS score decreases).

HMR is commonly used to study the excitability of spinal motor circuitry. In particular, HMR represents the neurophysiological correlate of the function of spinal inhibitory interneurons and of the descending pathways impairment in spasticity [49]. HMR alterations are therefore associated with spasticity and correlate, although nonlinearly, with MAS scores [50]. A higher ratio suggests higher corticospinal excitability subtending spasticity.

In the present trial, the primary endpoint with respect to the efficacy of MV was the proportion of patients achieving a minimal detectable change (which is a statistical estimate of the smallest amount of change that can be detected by a measure, corresponding to a noticeable change in the measure) of approximately a one-point decrease on the MAS as reported in the literature [51]. This minimal detectable change also reflects a clinically important difference [51]. Concerning SICI and HMR, there are no data available on the minimal detectable change. Nonetheless, we found in our previous work on Armeo-Power that a decrease in SICI and HMR of at least 15% represents a noticeable change [13].

### Secondary outcome measures

We measured the effects of training on the recovery in post-stroke hemiplegic patients as measured by Fugl-Meyer Assessment of the Upper Extremity (FMA-UE) [52–53]. Each item of motor function for the upper limb is scored on a 3-point ordinal scale (0 = cannot perform, 1 = performs partially, and 2 = performs fully; the total score ranges from 0 to 66).

Disability burden was assessed by the Functional Independence Measure (FIM)[54], which provides a measure for disability based on the International Classification of Impairment, Disabilities and Handicaps. FIM measures the level of a patient's disability and indicates how much assistance is required for the individual to carry out activities of daily living. Beyond the total FIM score, we assessed some subitems, i.e., eating, grooming, bathing, upper body dressing, lower body dressing, and toileting, which are related to self-care mainly involving upper limb function [55]. Each task is rated on a 7-point ordinal scale that ranges from 1 = total assistance (or complete dependence) to 7 = complete independence.

We also assessed mood and anxiety by Hamilton Rating Scale for depression (HRS-D) and Hamilton Rating Scale for anxiety (HRS-A), which are multiple-item questionnaires used to provide an indication of depression and anxiety and as a guide to evaluate recovery [56–59]. The higher the score, the more severe is the mood/anxiety impairment.

Kinematic properties of upper limb were quantified by measuring with the Armeo-Power device the passive range of movement (measured in degree) and the force (in Newton × meter) of the abduction–adduction, flexion–extension, intrarotation–extrarotation of the shoulder, and the flexion and extension of the elbow. Moreover, we measured the arm weight support offered by the Armeo-Power device to sustain the upper limb during exercise training and the device guidance force (that is, the assist-as-needed support provided by the robotic arm exoskeleton that automatically adapts the force exerted by the device itself to the patient’s capabilities to accomplish the movements required by the tasks (i.e., shoulder abduction, arm flexion, and elbow extension through different spaces).

Additionally, we measured the resting motor threshold (measured as % of TMS stimulator output) and the peak-to-peak MEP amplitude (in mV), which more broadly reflects the excitability of corticomotor projections during muscle relaxation [60] and the intracortical facilitation (ICF). This is a measure of cortical facilitation carried by interneurons within the primary motor cortex. Similar to SICI, some correlations have been found between spasticity and ICF, given that this is abnormally increased in pure spasticity [61]. Consequently, one would expect a weakening (i.e., a decrease) of ICF when spasticity improves (i.e., the MAS score decreases).

Finally, we quantified the root mean square value from the surface electromyography signals of all the vibrated (triceps brachialis, supraspinatus, and deltoid) and nonvibrated muscles (biceps brachii, latissimus dorsi, and pectoralis maior). Root mean square value quantifies and reflects the physiological activity in the motor unit during contraction, thus expressing a correlation between the contraction force and the root mean square value [62].

### Transcranial magnetic stimulation

Primary motor cortex excitability at rest was tested through monophasic TMS pulses delivered by a figure-of-eight coil (with an external loop diameter of 9 cm) wired to a high-power Magstim200 stimulator 2 [63]. During the experiments, EMG activity was continuously monitored by visual- auditory feedback (i.e., an oscilloscope with loudspeakers, which was placed in front of the subject) to ensure complete muscle relaxation. We first determined the resting motor threshold from biceps brachii muscle [64]. Then, we delivered 15 supra-threshold monophasic pulses (120% resting motor threshold), and the mean amplitude was calculated. SICI and ICF were determined according to the paired-pulse method described by Kujirai and colleagues [64]. The intensity of the conditioning stimulus was set at 70% of resting motor threshold. The intensity of the test stimulus was 120% resting motor threshold. Stimulus intensities were kept constant across the blocks of measurement. SICI and ICF were assessed at an ISI of 2 and 12 ms, respectively [65–71]. Fifteen trials were recorded for each ISI and randomly intermingled with 15 trials in which MEPs were elicited by the test stimulus alone. The peak-to-peak amplitude of the unconditioned MEP was taken as a measure of corticospinal excitability. Mean amplitude of the conditioned MEP was expressed as a percentage of the amplitude of the unconditioned MEP. The relative change in MEP amplitude induced by the conditioning stimulus characterized the strength of SICI and ICF.

### Hmax/Mmax ratio

The H-reflex and M-wave were recorded in the affected arm while the subject lay prone on a gurney with the shoulder abducted to 90°, palm facing up with the elbow slightly flexed. Bipolar Ag-AgCl surface electrodes (Nicolet Biomedical, Maddison, Wisconsin, USA) were applied in a belly-tendon montage over the spastic biceps brachii. The H-reflex was identified as a triphasic wave with a small initial positive deflection followed by a larger negative one. The maximum amplitudes of the H-reflex and the M-wave were measured from the peak of the positive to the peak of the negative deflections. The HMR was calculated by dividing the maximum amplitudes of the H-reflex by that of the M-wave.

### Surface electromyographic recording

Surface adhesive electrodes were applied on both vibrated and nonvibrated muscles, with a bipolar belly-tendon montage. Although the supraspinatus has not routinely been monitored through surface electrodes, a small window of access to the trapezius tendon exists (i.e., at the midpoint and two fingerbreadths superior to the scapular spine) [72]. In addition, we used a high-pass filtering [73], and the raw signals were amplified and filtered at 30–1000Hz (Neurolog System) (Digitimer Ltd, Welwyn Garden City, UK). Patients wore the robotic arm and were invited to align their upper limb to the scapular plane to the best of their ability, thus abducting, flexing, and extra-rotating the arm, and extending the elbow. The task was repeated 15 times. The subject had to perform such tasks without Armeo-Power support (i.e., the subject held only the weight of his arm). The electrical activity that is displayed in form of surface EMG signals is the result of neuromuscular activation associated with muscle contraction. The amplitude of EMG signal reveals is roughly proportional to the force exerted by the underlying muscle. To analyze the amplitude of surface EMG signal, we calculated the root mean square, that is the square root of average power of a signal for given period of time [74]. To this end, raw EMG data were full-wave rectified and processed using an algorithm with a 20 ms moving window. EMG with the greatest rectified and smoothed amplitude was quantified for a 2 sec period during each test. The data resulting from this period were utilized for analysis of each muscle test performed for normalization and each exercise.

### Statistical analysis

Descriptive statistics are given as mean ± standard deviation (SD) or median. The Shapiro-Wilk statistic was used to test the normality of the distribution of all variables; electrophysiological and kinematic measures were normally distributed (p>0.2), whereas clinical measures showed a non-normal distribution (p<0.05). Therefore, parametric and nonparametric statistics were used to describe changes from baseline (T0) to post-treatment (T1 and T2). One-way analysis of variance (ANOVA) for repeated measures or the Friedman test were performed depending on normal or non-normal distribution of the data, respectively. A pair-wise comparison was performed using the Wilcoxon signed-rank test to identify significant difference across time. Repeated measure ANOVAs, followed by Bonferroni correction for multiple comparisons for *post-hoc* analysis, were used to examine differences between the groups (two levels: A and B) over *time* (three levels: T0, T1, and T2). Clinical-demographic characteristics (age, gender, disease duration, and localization of brain lesion at magnetic resonance imaging) and kinematic factors (body weight support and device guidance force) were added in the ANOVA as covariates. Descriptive analysis was used to evaluate the effect size measures between the two groups (Cohen's d calculation).

We measured the incidence of subjects (namely, “responder patients”) exceeding a decrement of at least 1-point at the MAS from T0 to T2 (i.e., a minimal detectable change at the 95% confidence interval, according to literature data) [50–51] and a decrement of at least 15% at SICI and HMR (according to our previous work) [13]. To assess the difference between the two groups, we calculated the relative risk (RR) of an improvement when the patient is really treated with MV. A patient was considered improved when the minimal detectable change at T2 of the MAS score was a decrease of at least 1 point at the MAS and of at least 15% at the SICI and HMR (at the 95% confidence interval and according to the currently available data) [13,50–51,75]. Finally, clinical-electrophysiological correlations were evaluated by Fisher’s exact test.

Statistical analyses were carried out using Statview software (version 5; SAS Institute Inc., Cary, NC).

## Results

Twenty patients were recruited from January 2015 to June 2015. The recruitment was then stopped because all the patients who were treated at our rehabilitation unit had been evaluated to participate in the study. Baseline clinical-demographic characteristics (age, gender, disease duration, and localization of brain lesion at magnetic resonance imaging) were similar in both groups (Table 1).

**Table 1:** Clinical-demographic characteristics at baseline.

Parameter		A	B
Age (years)		66±5	67±4
Gender (M:F)		5:5	4:6
Disease duration (months)		5±2	6±2
MRI pattern (n. of patients)	1	2	2
	2	2	2
	3	2	4
	4	2	2
	5	2	0
MAS		3.4±0.9	3.2±0.8
FMA-UE		23±14	22±17
FIM (all items)		63±4	73±3
FIM (six items)		21±2	31±2
HRS-D		19±4	21±2
HRS-A		10±5	8±4

Legend: MAS Modified Ashworth Scale, FMA-UE Fugl-Meyer Assessment, FIM Functional Independence Measure, HRS-D Hamilton Rating Scale for depression, HRS-A Hamilton Rating Scale for anxiety, MRI number of patients with a lesion site at magnetic resonance imaging (1, cortical/subcortical fronto-parietal, 2, cortical/subcortical fronto-temporo-parietal, 3 cortical/subcortical parietal, 4 cortical/subcortical parieto-temporal, 5 subcortical).

All the patients showed a mild-to-severe upper limb motor impairment and disability burden, in parallel to low MEP amplitude and high SICI, ICF, and HMR values from the spastic biceps brachii (Table 2). Arm weight support and device guidance force were initially set at 40% and 80%, respectively, in both groups.

**Table 2:** Repeated results of primary clinical and electrophysiological outcomes.

	group	T0	T1	T2	Post-hoc T1	Post-hoc T2	d
MAS	A	3.4±0.9	2±0.6	3±0.6	<0.001	0.007	0.6
	B	3.2±0.8	2.4±0.7	3.2±0.5	0.3	0.4	
SICI (%)	A	80±2	51±2	50±3	<0.001	<0.001	0.7
	B	79±3	69±3	81±3	0.5	0.1	
HMR (%)	A	130±3	81±4	89±5	<0.001	<0.001	0.7
	B	131±3	96±4	128±3	0.3	0.5	

Legend: MAS Modified Ashworth Scale, SICI short intracortical inhibition, HMR H_max_/M_max_ ratio, NS non-significant.

### Primary outcomes

All the patients of group-A and three patients of group-B (30%) achieved the primary endpoint (namely, “responder patients”), i.e., a MAS reduction of at least 1 point and an HMR and SICI decrease of at least 15% (RR = 3.3; 95% confidence interval 1.29 to 8.59; p = 0.01). Primary outcome measures are summarized in Table 2. The repeated-measures analysis showed a significant interaction *time×group* for each primary outcome (p<0.001), thus indicating that there was a significant difference between the groups at T1 and T2.

The results show a significant reduction in MAS, SICI, and HMR at T1 and T2 only in group-A (p<0.001).

Concerning clinical-electrophysiological correlations (Fisher’s exact test), we observed that a greater decrease in MAS scoring was correlated to a greater SICI strengthening in the biceps brachii (Z = 2.8, p = 0.004).

### Secondary clinical outcomes

The patients of group-A showed a more evident clinical improvement than group-B patients, with significant FIM six items and FMA-UE increase, and HRS-D and HRS-A decrease. Secondary clinical outcome measures are summarized in Table 3.

**Table 3:** Repeated results of secondary clinical and electrophysiological outcomes.

	group	T0	T1	T2	Post-hoc T1	Post-hoc T2	d
FIM Six-items	A	21±2	26±3	25±2	<0.001	0.01	0.7
	B	31±2	33±2	32±1	0.2	0.3	
FMA-UE	A	23±14	37±8	26±6	0.001	0.007	0.4
	B	22±17	26±4	27±5	0.04	0.3	
HRS-A	A	10±5	7±2	7±2	0.001	0.001	0.7
	B	8±4	8±2	8±2	0.1	0.2	
HRS-D	A	19±5	11±3	11±3	0.001	0.001	0.6
	B	21±2	18±4	18±4	0.2	0.5	
MEP (mV)	A	0.41±0.1	0.5±0.1	0.52±0.1	0.001	0.007	0.8
	B	0.38±0.1	0.4±0.1	0.41±0.1	0.3	0.4	
ICF (%)	A	111±8	112±8	115±10	0.4	0.3	0.1
	B	109±8	109±7	110±8	0.1	0.2	

Legend: FIM Functional Independence Measure, FMA-UE Fugl-Meyer Assessment, HamD Hamilton Rating Scale for depression, HamA Hamilton Rating Scale for anxiety, MEP motor evoked potential, ICF intracortical facilitation, NS non-significant.

The repeated-measures analysis showed a significant interaction *time×group* for the six items of FIM (p<0.001), FMA-UE (p = 0.003), HRS-D (p = 0.02), and HRS-A (p = 0.001), thus indicating that there was a significant difference between the groups at T1 and T2.

However, a significant increase in FIM, and a decrease in HRS-D and HRS-A was found only in the group-A (p<0.001). FMA-UE increased in the group-A at T1 and T2 (p<0.001), whereas it augmented in group-B only at T1 (p<0.001).

As additional data, we observed a reduction in flexion muscle synergies (couplings of shoulder elevation movements with elbow flexion) in favor of extension muscle synergies (shoulder adduction/internal-rotation with elbow extension) in all patients of group-A and three subjects of group-B.

### Secondary electrophysiological outcomes

The patients of group-A showed a greater activation of vibrated muscles and a MEP amplitude increase than patients of group-B. Secondary electrophysiological outcome measures are summarized in Tables 3 and 4.

**Table 4:** Repeated results of secondary kinematic outcomes.

			group	T0	T1	T2	Post-hoc T1	Post-hoc T2	d
force (N×m)	E-fl/ex		A	1±0.1	1.5±0.1	0.8±0.1	0.4	0.3	0.1
			B	0.9±0.1	1.2±0.1	0.8±0.1			
	S-ab/ad		A	0.8±0.1	0.9±0.3	0.9±0.1	<0.001	<0.001	0.6
			B	0.7±0.1	0.8±0.2	0.7±0.4	0.03	0.5	
	S-fl/ex		A	2.3±0.1	5.5±0.4	3.9±0.2	0.2	0.2	0.1
			B	2.4±0.1	4±0.5	3±0.2			
	S-ir/er		A	2±0.1	6.3±0.1	5±0.1	0.3	0.3	0.1
			B	1.7±0.1	6±0.1	2±0.1			
ROM (deg)	E-fl/ex		A	46±4	76±5	61±4	0.5	0.2	0.1
			B	48±4	68±4	61±3			
	S-ab/ad		A	64±2	81±6	76±3	<0.001	0.03	0.6
			B	61±2	71±5	65±3	<0.001	0.3	
	S-fl/ex		A	69±4	82±4	79±3	0.5	0.5	0.1
			B	67±3	72±4	65±2			
	S-ir/er		A	72±3	81±10	77±5	0.2	0.5	0.1
			B	73±4	77±8	75±4			
AWS (%)			A	41±3	31±2	34±3	<0.001	<0.001	0.6
			B	39±3	33±2	38±3	0.01	0.2	
DGF (%)			A	81±3	60±2	66±2	<0.001	<0.001	0.4
			B	82±2	69±2	80±2	0.01	0.3	
RMS (μV)	non-vibrated	BB	A	114±12	120±13	118±12	0.1	0.2	0.1
			B	114±12	121±13	115±12			
		LD	A	79±6	88±7	85±6	0.5	0.1	0.1
			B	82±5	84±9	82±5			
		PM	A	80±7	86±8	84±4	0.4	0.4	0.1
			B	83±4	84±5	82±3			
	Vibrated	DE	A	123±8	162±15	145±15	<0.001	<0.001	0.8
			B	128±10	138±16	135±14	0.4	0.4	
		SS	A	48±5	76±8	62±8	<0.001	<0.001	0.8
			B	50±6	66±7	60±5	0.3	0.1	
		TB	A	79±6	112±9	98±5	0.002	0.009	0.4
			B	83±9	90±5	86±4	0.01	0.03	

Legend: ROM range of movement, RMS root mean square, AWS arm weight support, DGF device guidance force, E elbow, S shoulder, fl/ex flexion/extension, ab/ad abduction/adduction, ir/er intrarotation/extrarotation, BB biceps brachii, LD latissimus dorsi, PM pectoralis maior, DE deltoids, SS supraspinatus, TB triceps brachii, N·m Newton×meter, NS non-significant.

The repeated-measures analysis showed a significant interaction *time×group* for MEP amplitude (p = 0.002) and root mean square magnitude of deltoids (p = 0.01) and supraspinatus (p<0.001), thus indicating that there was a significant difference between the groups at T1 and T2. In particular, we found an increase of MEP amplitude and root mean square magnitude of deltoids and supraspinatus only in the group-A at T1 and T2 (p<0.001). Instead, the magnitude of root mean square magnitude of triceps brachii increased in both groups at T1 and T2 (p<0.001)

The strength of ICF and the magnitude of root mean square magnitude of non-vibrated muscles showed no significant changes.

### Secondary kinematic outcomes

The patients of group-A showed a greater kinematic amelioration (consisting of an increase in passive range of motion and force in shoulder abduction and adduction movements, and in reduced arm weight support and device guidance force) than the patients of group-B (Table 4). The repeated-measures analysis showed a significant interaction *time×group* for arm weight support (p<0.001), device guidance force (p = 0.006), and force (p<0.001) and passive range of movement (p<0.001) of shoulder abduction and adduction, thus indicating that there was a significant difference between the groups at T1 and T2.

Specifically, the increase of force and range of movement of shoulder abduction and adduction, and the decrease of arm weight support and device guidance force were significant in group-A at T1 and T2 (p<0.001), whereas such changes were significant in group-B only at T1. The remaining kinematic parameters showed no changes.

To investigate whether MAS change (as main primary outcome measure) induced by MV could be affected by arm weight support and device guidance force, we calculated an ANOVA using these factors as covariates. There were no interactions among arm weight support, device guidance force, and MV in both the groups (*group×arm-weight-support×device-guidance-force* p = 0.9).

## Discussion

The data of our pilot study suggest the usefulness of focal MV when combined with robotic neurorehabilitation in managing upper limb spasticity in chronic stroke patients. In fact, MV induced a MAS decrease, paralleled by an HMR decrease and a SICI strengthening in all the patients of group-A, who thus achieved the primary outcome. Moreover, the MAS decrease correlated significantly with SICI potentiation. Finally, MV strengthened the amelioration of the other outcomes yielded by the Armeo-Power alone and determined a duration of Armeo-Power aftereffects up to 1 month, as compared to Armeo-Power delivered alone. Altogether, these data suggest that the improvement in spasticity (namely, MAS reduction) induced by the association between motor training and MV may depend on a modulation of motor cortex and spinal excitability, i.e., an increase of the inhibitory output from motor cortex to spinal level, as suggested by the SICI increase and the HMR decrease. In fact, Armeo-Power alone did not influence MAS, SICI, or HMR substantially (except in three patients). Nonetheless, we have to be cautious in interpreting our data concerning the efficacy of focal MV when combined with robotic neurorehabilitation, given the underpowered nature of the study. Although promising, our data ought confirmation by further large sample studies.

### Putative mechanisms of spasticity reduction

There are some conflicting reports in the literature concerning the improvement in spasticity sustained by MV and Armeo-Power practiced alone [76–77]. Our data suggest that MV combined with robotic neurorehabilitation may improve spasticity in post-stroke patients, probably in keeping with the principles of associative plasticity [9–12,23,32–33]. In fact, MV allowed for boosting corticospinal excitability at both the cortical and spinal level (i.e., a clear modulation SICI and HMR) as compared to Armeo-Power delivered alone. These effects may depend on a direct entrainment of muscle spindle Ia-afferent firing up to 80Hz, which may be sensitive to MV protocol [16–17]. Of note, we applied an intensity of vibration below the threshold for eliciting tonic vibratory reflex or inducing movements. This may have increased antagonist muscle activation and reduced agonist activation, probably by harnessing mechanisms of reciprocal inhibition, presynaptic inhibition, and changes in the intrinsic regulation of transmitter release from the Ia-afferents of spastic muscles at spinal level, besides variations of the intrinsic biomechanical and electrophysiological properties of the target muscles [23–27,33]. Nonetheless, some studies have shown that MV of spastic agonist muscle also led to a spasticity decrease, probably owing to post-activation depression phenomena, an increase of reflex threshold, or a co-contraction decrease [78–80]. In addition, MV effects may also depend on the characteristics of the MV device itself [23–27,33].

The amount of proprioceptive information from muscle and joint receptors that reaches the sensory-motor cortices during MV may also have an important role [22]. Indeed, the MV of antagonist muscles led to a primary motor cortex excitability increase and SICI potentiation in biceps brachii muscle (i.e., spastic agonist muscle). These findings agree with previous functional neuroimaging studies suggesting that MV activates primary sensory and motor areas (beyond premotor, supplementary motor, and cingulate cortices) [81]. Hence, in analogy with premotor-motor facilitation, the effects on SICI could depend on a modulation of bidirectional connections linking premotor and motor cortices sustaining MEP amplitude increase.

Notably, the Armeo-Power also offers a considerable amount of sensory input. In fact, it has been demonstrated that primary motor cortex and supplementary motor area are activated during a sensory stimulation using passive cyclical joint movements [82]. However, these passive movements activate the joint and cutaneous receptors more than the Ia-afferents, which are essential for primary motor cortex proprioceptive activation [83–84]. Thus, the prominent involvement of Ia-afferents could account for the stronger modulation of primary motor cortex excitability following the Armeo-Power paired with real MV in comparison to the Armeo-Power paired with sham-MV. In addition, proprioceptive stimuli modulate spinal reflexes more than the exteroceptive stimuli. Finally, the cortical processing of such information may in turn modulate spinal reflexes through cortico(-brainstem)-spinal inhibitory pathways onto spinal Ia-dependent inhibitory interneurons [83–84].

### Secondary outcomes

The combined approach also led to a greater improvement in mood, anxiety, and upper limb motor function (FMA-UE increase), as compared to Armeo-Power practice alone. We may argue that MV plays a key role in improving the gain in motor performance yielded by Armeo-Power practice, regardless of spasticity. This gain in motor performance may in turn improve mood and reduce anxiety. MV can recruit complex networks encompassing premotor-sensory-motor areas [85], thus probably enhancing movement planning, favoring the recruitment of perilesional and neighboring areas, and improving the feedback control of tracking movements, the volume of the available workspace, the movement smoothness, and the inter-joint coordination (which all represent targets of Armeo-Power practice). This may also reduce the abnormal muscle activation coupling [86–91], of which we observed a reduction of flexor synergies in favor of extensor synergies in all the patients of group-A and in three subjects of group-B. The decrease in pathologic compensatory strategies may have also contributed to the clinical amelioration. In fact, compensatory strategies have an immediate benefit on daily life activity, but they also have a negative impact on the quality of movement performance and limit the long-term prognosis owing to the learned disuse phenomenon [86–91].

### Study limitations

The main limitation of our pilot study consists of its underpowered nature. In fact, the required sample size, based on detecting the proportion of patients achieving a minimal detectable change of approximately a one-point decrease on the MAS and a decrease in SICI and HMR of at least 15% (two-tailed test, α level of 0.05, and 80%power), was 30 individuals per group. This limits the significance of multiple comparisons we made, as the required sample size increases linearly with the logarithm of the number of comparisons made [92–93]. Even though pilot-study guidelines report that stage-2 clinical rehabilitation pilot studies on physical and cognitive interventions, such as ours, can begin with a convenience sample of at least six participants, we have to be cautious about the inferences that can be drawn from tis underpowered study [94]. Larger samples and crossover studies are necessary to confirm our promising findings.

Other limitations of our study consisted of the lack of a longer follow-up and the relatively high variability of FMA-UE scoring in both groups (slightly more evident in group-B). Such high variability could account for the milder responsiveness of group-B patients to Armeo-Power training alone, while MV allowed group-A patients to meet primary and several secondary outcomes for a longer period, thus strengthening Armeo-Power aftereffects.

Additionally, the improvements observed in those three patients in group-B (“responder” patients, as those belonging to group-A) may question the possibility that the clinical-electrophysiological changes were due to the combination of the interventions, given that they could rather depend on the intensive and repetitive motor activity during Armeo-Power training. However, these group-B “responder” patients had lower FMA-UE and MAS scoring as compared to the other group-B “nonresponder” subjects. Nonetheless, the group-B “responder” patients (as well as the other group-B “nonresponder” subjects) neither showed the corticospinal excitability modulation shown by group-A, nor maintained their outcome improvement at T2 (as instead the patients of group-A did). These issues may confirm that MV is consistently able to strengthen and prolong Armeo-Power effects, probably through both spasticity reduction and corticospinal excitability modulation, beyond primarily facilitating spasticity reduction.

Finally, FIM values are a few below those available in the literature, which however come from patients with a more varying disease duration and mixed stroke etiology than our patients [95–100].

## Conclusions

Our pilot study suggests that MV combined with Armeo-Power may consistently reduce upper limb spasticity, and strengthen and lengthen the Armeo-Power effects regarding upper limb motor function, mood, and the disability burden. The stronger effect of the combined approach on spasticity and upper limb functions may depend on a sort of associative plasticity between the two coupled trainings, which could have reshaped corticospinal plasticity with a consequent reduction of segmental excitability at the spinal level and an entrainment of recovery processes at the cortical level.

Although larger samples and crossover studies are necessary to confirm our promising findings, we may suggest that MV could be usefully harnessed to increase the functional outcomes obtained by using Armeo-Power, and to improve upper limb spasticity and functions in post-stroke patients.

## Suppliers list

a Armeo-Power; Hocoma AG, Switzerland, Industriestrasse 4 CH-8604 Volketswil; Tel. +41434442200, Fax +41434442201; info@hocoma.com; www.hocoma.com.
b Vibraplus; a circle s.p.a., via Ferrara 21–40018 San Pietro in Casale (BO) Italy; tel.: +39051817550—fax: +39051811993.

## Supporting information

S1 Table CONSORT checklist. (DOC)

S1 File Trial study protocol in English language. (DOC)

S2 File Trial study protocol in Italian language. (DOC)
