# Supplementary material for: Is two better than one? Muscle vibration plus robotic rehabilitation to improve upper limb spasticity and function: A pilot randomized controlled trial
Source: PLoS One. 2017 Oct 3;12(10):e0185936. doi: 10.1371/journal.pone.0185936 (PMC5626518; doi:10.1371/journal.pone.0185936)
Supplement: S1 File — (DOC) [file pone.0185936.s002.doc]

Title: “Effect of non-invasive neurostimulation on motor performance of patients with acquired neurological disorders”.

Study desing: Observational

Target disease: Acquired neurological pathology

Rationale: Acquired neurological disorders raise severe and disabling consequences on functional outcomes in relation to dexterity and walking. Rehabilitation is an important part of patient treatment. There are innumerable potentially useful therapeutic options for motor recovery in the field of rehabilitation, especially neuro-robotics. In addition, there are several studies on new methods in support of rehabilitation, such as electrophysiological stimulation by using transcranial magnetic (TMS) or electric (TES) stimulation (Langhorne et al ., 2011). These innovative, non-invasive, and substantially safe methods are useful to better understand the recovery mechanisms of cortical lesions, such as those stroke-induced in the motor areas, particularly. The use of such methods is also found to be important in promoting the recovery of impaired motor function by modifying cortical excitability, even several hours after their application (Liepert et al., 2000). This could be useful to favor motor performance and functional recovery. Recovery from brain lesion has distinct temporal phases (Rossini et al., 2003). The activation of intra-hemispherical and inter-hemispheric neuroplastic mechanisms plays a key role in the recovery (Feydy et al., 2002). Specific mechanisms can be studied and modulated through TMS and/or TES, and are therefore potentially exploitable in rehabilitation to improve functional recovery. This depends heavily on the quality of rehabilitation and, in particular, on the standardization of treatment. Robotic devices, such as the Lokomat (Hocoma Inc, Rockland, MA) and the Armeo (for the lower and upper limbs, respectively) have been introduced to improve these motor recovery. Robots are cooperative controllers with the patient, i.e., they take into account patient intentions and efforts rather than imposing any predefined movement. With this system, patients can repeat the movements many times and more precisely than with classical physiotherapy. It has been shown that the patients who undertake a robot-assisted rehabilitation improve in motor performance and maintain motor function gain during the chronic phase. This is probably due to the fact that robot-assisted movement repetition has better symmetry and are more physiological. In keeping with these issues, combining electrophysiological methods capable of modulating synaptic plasticity and neuro-robotic motor training could further improve the rehabilitative outcomes of the patient with acquired neurological pathology, including stroke.

Objectives: It is well known that a hemispheric lesion changes the balance between excitation and inhibition between the hemispheres, in a variable way over time, expressed as a down-regulation of activity in the affected hemisphere and an up-regulation of the activity in the unaffected hemisphere and in the perilesional areas. This up-regulation can interfere with functional recovery, motor performance, and rehabilitation training. The correctly timed and regional application of electrophysiological methods can appropriately re-balance this disequilibrium. The ease of application of non-invasive and painless methods such as TMS and TES, and their ability to modulate cortical excitability without interrupting brain activity, make such methods particularly suitable for a combination with neuro- robotics. Therefore, the aim of our study is to demonstrate the effectiveness of such methods applied before, during, or after the rehabilitation session by using Lokomat or Armeo, to improve motor performance in patients with limb paresis following acquired neurological pathology

Study design: This is an observational study that will involve IRCCS Centro Neurolesi "Bonino-Pulejo" in Messina, Italy. After having obtained the informed consent, 30 patients will be recruited according to the under-defined criteria. They will be evaluated clinically and randomly divided into two groups. Each group will participate in a motor rehabilitation program consisting of ten sessions of 30 minutes of robotic training, five days a week (Monday to Friday) for two consecutive weeks. A group will perform non-invasive neuromodulation treatment, the other a sham non-invasive neuromodulation treatment. Noninvasive neuromodulation treatment may consist of TMS, TES, muscle vibration, or other non-invasive non-invasive methods. Neuromodulation protocols will be carried out in accordance with current guidelines in force (Zaghi et al., 2010; Quartarone et al., 2006; Jayaram et al. 2008; Stefan et al., 2008; Ridding et al. 2000, 2001; Pyndt e Ridding, 2004). Patients will be evaluated prior to treatment (T0), immediately after treatment (T1, primary endpoint), and two weeks after the end of treatment (T2), by the same examiner who will be unaware of the treatment received by the patient. Primary outcome measure evaluation will be performed using specific test (including six minute walking test and 10m walking test). Secondary outcome measurements will include, among other, electrophysiologic measures of corticospinal excitability via TMS, the Fugl-Meyer Assessment, the Rivermead Mobility Index, the Functional Independence Measure, the Modified Ashworth Scale, and the SF-36 questionnaire.

Inclusion Criteria: Age <75 years; Mini Mental State Examination score> 24; to be upright without aids for at least 5 minutes; Ability to walk alone for at least 15m, even with the use of aids for walking (crutch and / or orthosis).

Exclusion criteria: Positive epilepsy history; Electroencephalographic activity with epileptic elements; Metal or electrical devices at cranial level; Previous neurosurgery; Intake of antiepileptic, neuroleptic drugs, benzodiazepines, antidepressants, dopaminergic drugs; hemi-hypoesthesia; Presence of vestibular disorders or paroxysmal vertigo; Severe cognitive deficiency; Other neurological disorders or orthopedic conditions involving the limbs; Serious cardiovascular disease; Any type of rehabilitation treatment within the three months prior to the beginning of the study.

Statistical Analysis: The Kruskal-Wallis test will evaluate the homogeneity of the groups. Friedman's test variations will assess performance of each group in different sessions. The Wilcoxon signed test scores will measure pre/post-treatment and pre-treatment/follow-up scores for different outcome measures in each patient group. The U-test Mann-Whitney will compare the effect of treatment in the 2 groups. For this reason, the differences in performance between post- and pre-treatment and between follow-up and pre-treatment for all endpoints will be evaluated. The α-level for significance will be p <0.05. Bonferroni correction for multiple comparisons at p <0.025. Statistical analysis will use SPSS for Windows statistical package version 16.0 (SPSS Inc., Chicago, IL, US).
