# Supplementary material for: Is two better than one? Muscle vibration plus robotic rehabilitation to improve upper limb spasticity and function: A pilot randomized controlled trial
Source: PLoS One. 2017 Oct 3;12(10):e0185936. doi: 10.1371/journal.pone.0185936 (PMC5626518; doi:10.1371/journal.pone.0185936)
Supplement: S2 File — (DOC) [file pone.0185936.s003.doc]

Titolo dello studio: “Effetto della neurostimolazione non invasiva sulle performance motorie del cammino robot-assistite in pazienti con patologie neurologiche acquisite”.

Tipo di Studio: Osservazionale

Malattia target: patologia neurologica acquisita

Razionale: Le patologie neurologiche acquisite rappresentano una problematica con esiti disfunzionali spesso gravi ed invalidanti, specie in relazione alla manualità ed il cammino, e la riabilitazione è una parte importante del trattamento del paziente. Vi sono innumerevoli opzioni terapeutiche in ambito riabilitativo potenzialmente utili per il recupero motorio, in particolare la neuro-robotica. Inoltre vi sono diversi studi su nuove metodologie in supporto delle metodiche riabilitative quali, in particolare quelle elettrofisiologiche come la stimolazione magnetica (TMS) e/o elettrica (TES: a corrente continua –tDCS-, alternata –tACS-) transcranica (Langhorne et al., 2011). Queste ultime metodiche, non invasive e sostanzialmente sicure, si stanno rivelando di notevole aiuto per meglio comprendere i meccanismi di recupero delle funzioni corticali lese, come nello stroke ad esempio, in particolare in ambito motorio. L’uso di tali metodiche si sta rivelando inoltre importante nel favorire, attraverso specifiche metodiche, il recupero della funzionalità motoria lesa, essendo in grado di perturbare l’eccitabilità corticale anche diverse ore dopo la loro applicazione (Liepert et al., 2000). Ciò potrebbe essere utile per favorire la performance motoria ed il recupero funzionale. Tale recupero ha fasi temporali distinte (Rossini et al., 2003). L’attivazione dei meccanismi di neuroplasticità intra-emisferica ed inter-emisferica giocano un ruolo chiave nel recupero della funzione lesa (Feydy et al., 2002). I meccanismi specifici possono essere studiati e modulati attraverso la TMS e/o TES, e quindi sono sfruttabili potenzialmente in chiave riabilitativa per migliorare il recupero funzionale. Il recupero funzionale dipende fortemente dalla qualità della riabilitazione ed in particolare dalla standardizzazione del trattamento. Per migliorare questi problemi sono stati introdotti dei dispositivi robotici, come il Lokomat (Hocoma Inc, Rockland, MA) e l’Armeo (per gli arti inferiori e superiori, rispettivamente). I robot sono controllori cooperativi col paziente, nel senso che tengono conto dell’intenzione e degli sforzi del paziente piuttosto che imporre qualsiasi movimento predefinito. Con questo sistema, i pazienti possono ripetere i movimenti più spesso e più precisamente che con la fisioterapia classica. Si è visto che i pazienti che praticano una riabilitazione del cammino robot-assistita migliorano le prestazioni deambulatorie e le mantengono al follow-up durante la fase cronica. Ciò è probabilmente dovuto al fatto che la ripetizione robot-assistita dell’andatura permette ai pazienti di praticare un ciclo del passo completo, con migliore simmetria e walking fisiologico, on un ottimale mobilizzazione della arto superiore/mano. Sulla base di quanto sinora detto, coniugare le metodiche elettrofisiologiche in grado di modulare la plasticità sinaptica, soprattutto relativa alla connettività interemisferica, col training motorio neuro-robotico potrebbe migliorare ulteriormente l’outcome riabilitativo del paziente con patologia neurologica acquisita, incluso lo stroke.

Obiettivi: E’ ben noto che una lesione emisferica in area motoria altera l'equilibrio tra eccitazione e inibizione tra gli emisferi, in modo variabile nel tempo, espresse come down-regulation dell'attività nell'emisfero lesionale, up-regulation dell’attività nell'emisfero sano, o sovra-regolazione delle attività nelle regioni perilesionali. Tutto ciò può interferire con il recupero funzionale, la performance motoria ed il training riabilitativo. La corretta applicazione delle metodiche elettrofisiologiche, può opportunamente bilanciare tale dis-regolazione. La facilità di applicazione delle metodiche non invasive e non dolorose come la TMS e la TES (tDCS e tACS) e la capacità di modulare l’eccitabilità corticale senza interrompere l’attività cerebrale rendono tali metodiche particolarmente adatte per la combinazione con la riabilitazione neuro-robotica. Pertanto obbiettivo del nostro studio è quello di dimostrare in pazienti con patologia neurologica acquisita con limitazione dell’arto superiore, l’efficacia di tali metodiche applicate prima-durante-dopo la sessione di trattamento riabilitativo attraverso l’uso del dispositivo di neuro-riabilitazione robotica (Hocoma Inc, Volketswil, Switzerland), nel migliorare la performance motoria.

Disegno dello studio: Si tratta di uno studio osservazionale che coinvolgerà L’Istituto IRCCS Centro Neurolesi “Bonino-Pulejo”. Verranno reclutati, dopo aver ottenuto il consenso informato, 30 pazienti selezionati secondo i sotto-definiti criteri. Verranno valutati clinicamente e suddivisi in modo random in due gruppi; ogni gruppo parteciperà ad un programma di riabilitazione motoria composta da dieci sedute di 30 minuti di training robotico, cinque giorni alla settimana (da lunedi a venerdì) per due settimane consecutive. Un gruppo effettuerà in associazione un trattamento di neuromodulazione non-invasiva real ed uno sham. Il trattamento di neuromodulazione non invasiva consisterà di TMS, TDCS, vibrazione muscolare, o altre metodiche similari non-invasive. I protocolli di neuromodulazione verranno effettuati in accordo con le correnti linee guida vigenti in materia (Zaghi et al., 2010; Quartarone et al., 2006; Jayaram e coll. (2008) Stefan et al., 2008) Ridding et al., 2000, 2001; Pyndt e Ridding, 2004).

I pazienti saranno valutati prima del trattamento (T0), immediatamente dopo il trattamento (T1, endpoint primario), e due settimane dopo la fine del trattamento (T2), dallo stesso esaminatore non a conoscenza del trattamento ricevuto dal paziente. La valutazione dei pazienti avverrà attraverso l’uso test specifici (six minute walking test e del 10m walking test) ed elettrosifiologici tramite la TMS per studiare l’eccitabilità corticospinale. Misure secondarie saranno effettuate attraverso il Fugl-Meyer Assessment, Rivermead Mobility Index, Functional Independence Measure, Modiﬁed Ashworth Scale ed il questionario SF-36.

Criteri d’inclusione: Età <75 anni; Mini Mental State Examination score >24; capacità di mantenere la stazione eretta senza ausili per almeno 5’; capacità di camminare autonomamente per almeno 15m anche con uso di ausili per la deambulazione (stampella e/o ortesi). Criteri di esclusione: Anamnesi positiva per epilessia; attività elettroencefalografica con grafoelementi epilettiformi; componenti metalliche o dispositivi elettrici a livello cranico; precedenti neurochirurgici; assunzione di farmaci antiepilettici, neurolettici, benzodiazepine, antidepressivi, dopaminergici; emi-ipoestesia; presenza di disturbi vestibolari o di vertigine parossistica; grave deficit cognitivo; altre turbe neurologiche o condizioni ortopediche che coinvolgano gli arti inferiori; gravi malattie cardiovascolari; qualsiasi tipo di trattamento riabilitativo nei tre mesi precedenti l'inizio dello studio.

Analisi Statistica: Il test Kruskal–Wallis valuterà l’omogeneità pre-studio dei gruppi. Il test di Friedman le variazioni di performance nelle differenti sessioni in ogni gruppo. Il test Wilcoxon signed ranks i punteggi pre/post-trattamento e quelli pretrattamento/follow-up per le diverse misure di outcome in ogni gruppo di pazienti. L’U-test Mann–Whitney comparerà l’effetto del trattamento nei 2 gruppi. Per tale motivo saranno valutate le differenze di performance tra post- e pre-trattamento e tra follow-up e pre-trattamento per tutti gli endpoint. L’α-level per la significatività sarà p<0.05. Il correttivo di Bonferroni per comparazioni multiple a p< 0.025. Per l’analisi statistica sarà utilizzato il programma SPSS per Windows statistical package, version 16.0 (SPSS Inc., Chicago, IL, US).
